# Chrysophanol: A Natural Anthraquinone with Multifaceted Biotherapeutic Potential

**DOI:** 10.3390/biom9020068

**Published:** 2019-02-18

**Authors:** Prateeksha, Mohd Aslam Yusuf, Brahma N. Singh, Surya Sudheer, Ravindra N. Kharwar, Saba Siddiqui, Ahmed M. Abdel-Azeem, Leonardo Fernandes Fraceto, Kavya Dashora, Vijai K. Gupta

**Affiliations:** 1 Department of Biosciences, Integral University, Lucknow-226026, Uttar Pradesh, India; pratikschemistry@gmail.com; 2 Herbal Nanobiotechnology Lab, Pharmacology Division, CSIR-National Botanical Research Institute, Lucknow-226001, Uttar Pradesh, India; 3 Department of Bioengineering, Integral University, Lucknow-226016, Uttar Pradesh, India; aslambio@iul.ac.in; 4 Department of Chemistry and Biotechnology, ERA Chair of Green Chemistry, Tallinn University of Technology, 12618 Tallinn, Estonia; soorya.m.sudheer@gmail.com; 5 Centre of Advanced Study in Botany, Institute of Science, Banaras Hindu University, Varanasi-221005, Uttar Pradesh, India; rnkharwar@gmail.com; 6 Integral Institute of Agricultural Science and Technology (IIAST), Integral University, Lucknow-226026, Uttar Pradesh, India; 27.sabasiddiqui@gmail.com; 7 Botany Department, Faculty of Science, University of Suez Canal, Ismailia 41522, Egypt; ahmed_abdelazeem@science.suez.edu.eg; 8 Institute of Science and Technology of Sorocaba, São Paulo State University–Unesp, Sorocaba–São Paulo 18087-180, Brazil; leonardo.fraceto@unesp.br; 9 Centre for Rural Development and Technology, Indian Institute of Technology Delhi, Hauz Khas, New Delhi 110016, India; Kavya.Dashora@rdat.iitd.ac.in

**Keywords:** anthraquinone, chrysophanol, pharmacology, pharmacokinetics, toxicity

## Abstract

Chrysophanol is a unique anthraquinone having broad-spectrum therapeutic potential along with ecological importance. It is the first polyketide that has been reported to be biosynthesized in an organism-specific manner. The traditional Chinese and Korean medicinal systems provide evidence of the beneficial effects of chrysophanol on human health. The global distribution of chrysophanol encountered in two domains of life (bacteria and eukaryota) has motivated researchers to critically evaluate the properties of this compound. A plethora of literature is available on the pharmacological properties of chrysophanol, which include anticancer, hepatoprotective, neuroprotective, anti-inflammatory, antiulcer, and antimicrobial activities. However, the pharmacokinetics and toxicity studies on chrysophanol demand further investigations for it to be used as a drug. This is the first comprehensive review on the natural sources, biosynthetic pathways, and pharmacology of chrysophanol. Here we reviewed recent advancements made on the pharmacokinetics of the chrysophanol. Additionally, we have highlighted the knowledge gaps of its mechanism of action against diseases and toxicity aspects.

## 1. Introduction

Natural products were one of the key sources of therapeutic agents for thousands of years, however, the application of bioactive natural metabolites in traditional medicines and drug discoveries is still alive and well [1,2,3]. An array of secondary metabolites is produced by living organisms under different conditions, which are beneficial to the organisms and have a variety of applications for humans [4]. Anthraquinones is one of secondary metabolites that are produced by various plants and are applied in a wide range of applications, for example, as coloring agents in the food and textile industries and as therapeutic agents for various diseases [5,6]. They are derived from 9,10-anthracenedione. Addition of hydroxyl (-OH), methyl (-CH_3_), carboxyl (-COOH), and methoxyl (-OCH_3_) groups to 9,10-anthracenedione results in the formation of different anthraquinone derivatives, which possess a broad-spectrum of medicinal properties [6]. Chrysophanol is a 1,8-dihydroxy-3-methyl derivative of the 9,10-anthracenedione ring. This compound is widely distributed in several organisms, including plants, microbes, and insects.

Chrysophanol was first reported from *Rheum rhabarbarum*, a herbaceous perennial plant belonging to the Polygonaceae family [6]. To date, it is known to be present in various families, such as Polygonaceae, Rhamnaceae, Fabaceae, Liliaceae, Asphodelaceae, Buphorbiaceae, Meliaceae, Podocarpaceae, Picramniaceae, and Hemerocallidaceae [7,8]. It is considered as a quality control marker in some species of the genus, *Cassia*, belonging to Fabaceae [7]. However, its occurrence is rare in insects, where it is produced as a defensive chemical [9]. In fungi, chrysophanaol was first reported from *Penicillium* *islandicum* Sopp (ATCC 28431) [10]. It has been extensively explored in endosymbiotic fungi of marine organisms and plants. Importantly, chrysophanol is a major component of those plant extracts which have been utilized in many traditional Chinese medicines (TCM); for example, Quyu Qingre granules are used in blood stasis syndrome [11], Dahuang Fuzi decoction is used in chronic kidney disease [12], Da-cheng-Qi decoction is used in constipation, Yiqing capsules are used in inflammation [13], sososo is used in obesity [14], Yin Chen Hao Tang is used in acute hepatitis [15], Sanhuang is used in external injury [16], San-Huang-Xie-Xin-Tang is used in hypotension and gastric protection [17], and Masiningan is used in diabetes [18]. Chrysophanol is also present in many traditional Korean medicines (TKM); for example, in Ganweiqitong tablets used for obesity [19]. The antifungal effect of chrysophanol was reported in 1877 [20].

The therapeutic efficacy of chrysophanol as evidenced through the effects of TCM and TKM prompted researchers to verify it through in-vitro and in-vivo assays. Over the years, several lines of scientific investigations have confirmed the beneficial biological properties of chrysophanol, including its anticancer, antiviral, anti-diabetic, anti-inflammatory, antiprotozoal, hypolipidemic, hepatoprotective, neuroprotective, antiulcer, and anti-obesity effects. Biosynthetically, chrysophanol is a unique anthraquinone. It is produced via the polymalonate pathway (PMA) in fungi and via shikimate and PMA pathways in plants [21]. Several studies have been performed to verify the hypothesis as to whether the folding of the octaketide chain is organism specific. The folding of the octaketide chain was found to vary in an organism dependent manner: The “F” pattern was found to be present in fungi, insects, and plants whereas the “S” pattern was present in bacteria [22]. These differences in the folding patterns are unique in nature. In the present article, we have discussed the natural sources of chrysophanol, its biosynthesis, pharmacological and pharmacokinetic properties. The recent applications of chrysophanol are also discussed, which should help in guiding future research. In this review, the relevant information on chrysophanol (anthraquinones) was gathered from scientific databases including Google Scholar, Web of Science, SciFinder, ScienceDirect, PubMed, and Wiley Online Library. Information was also obtained from online databases, books, and Ph.D. theses.

## 2. Sources 

Chrysophanol is a tricyclic aromatic quinone, distributed across the plant and animal kingdoms, as well as in the microbial world. As of date, it has been reported in 14 genera from different families and in more than 65 species belonging to different genera (Table 1). It occurs in specific parts of plants, such as leaves, roots, rhizome pods, flowers, and bark. Its presence has been detected in approximately 29 species of *Cassia* genus belonging to Fabaceace. Two genera, *Rumex* and *Rheum*, belonging to Polygonaceae are the main sources of chrysophanol, which dominantly occurs in the roots and rhizomes of these plants [23,24]. The presence of chrysophanol has also been documented in the bark of some species of *Rhamnus*, a member of Rhamnaceae. A vast diversity of fungal mycelia is considered as a major reservoir of anthraquinones [25], but chrysophanol has been registered in very few endosymbiotic fungi from marine organisms and plants. Seven fungal families, namely Pleosporaceae, Dothideomycetes, Trichocomaceae, Cortinariaceae, Didymellaceae, Montagnulaceae and Hypocreaceae, have been reported to contain chrysophanol [26]. *Trichoderma harzianum* Strain Th-R16 has been reported to be enriched in chrysophanol [27]. The production of chrysophanol for competitive survival has been reported in *Trichoderma polysporum*, grown along with basidiomycete fungi, such as *Fomes annosus* [28].

A few lower plants, particularly lichens, such as *Asahinea chrysantha* belonging to Parmeliaceae, also efficiently synthesize a wide range of anthraquinones [76]. However, these compounds have not been explored much in prokaryotes. It was also reported to be present in the shield of some insects, which is a waxy layer used for defense; insects synthesize it as an adaptation against predators [9]. Members of only two families of insects, *Adelgidae* and *Chrysomelidae*, produce chrysophanol [9,76]. Besides, chrysophanol was also reported in the ethanolic extract of bee propolis (bee glue), a sticky resinous material collected by honeybees to architect and insulate their hives as well as to protect the hive from microbial (fungi and bacteria) growth [76].

## 3. Chemistry

Chrysophanol is an anthracene derivative with two ketone groups attached to the central benzene ring. It is also known as chrysophanic acid. The molecular formula of chrysophanol is C_15_H_10_O_4_, the molecular weight is 254.2 g/mol, and the melting point is 196 °C. It is a crystalline solid, occurring as golden yellow or brown powder, and exhibits maximum UV absorption at 225, 257, 277, 287 and 428 nm. The solubility of chrysophanol in water is poor; the aqueous solution is yellow but turns red on the addition of an alkali or concentrated sulfuric acid (https://pubchem.ncbi.nlm.nih.gov/search/#query=chrysophanol). Electrospray ionization mass spectrometer (ESI-MS) spectrum of chrysophanol exhibits one peak at 252.9 of [M − H]^−^ in negative ion scan mode while it is at 255 [M + H]^+^ in positive mode [77,78]. In MS/MS spectrum, The chrysophanol identified by base peak of 254 followed by second top peak of 255, and third highest peak of 226 of a stable molecule ion which is produced by removal of carbonyl group from the C-10 position because the C-9 is involved in the intramolecular hydrogen bonding with the α-hydroxyl groups at C-8 and C-1 [77]. The mass spectrum of chrysophanol is documented in main lib with NIST no. 112,526 (https://pubchem.ncbi.nlm.nih.gov/search/#query=chrysophanol). The 1H nuclear magnetic resonance (NMR) data of chrysophanol exhibited following peaks: 1 H-NMR (400 MHz, DMSO-d6) δ: 11.96 (1H, s, C1-OH), 11.86 (1H, s, C8-OH), 7.80 (1H, dd, J = 8.4, 7.6 Hz, C6-H), 7.71 (1H, d, J = 7.6 Hz C5–H), 7.55 (1H, d, J = 0.8 Hz, C4–H), 7.38 (1H, d, J = 8.4 Hz, C7–H), 7.22 (1H, d, J = 0.8 Hz, C2–H), 2.44 (3H, s, -CH3); and the 13 NMR data of chrysophanol displayed: 13C-NMR (100 MHz, DMSO-d6) δ: 191.4 (C-9), 181.3 (C-10), 161.4 (C-8), 161.1 (C-1), 149.0 (C-3), 137.2 (C-6), 133.2 (C10a), 132.8 (C-4a), 124.2 (C-2), 123.9 (C-7), 120.4 (C-4), 119.2 (C-5), 115.7 (C-8a), 113.6 (C-9a) 21.6 (-CH3) [79]. The functional group arrangement (methyl and hydroxyl) of chrysophanol on the anthraquinone ring is successfully passed form Lipinski’s rule of five or Pfizer’s rule of five which is extensively used to extract and evaluate the druglikeness of molecules. According to the rule, an idea drug should not have more than five hydrogen bond donors and should not have more than 10 hydrogen bond acceptors, and the molecular weight of the drug should be less than 500 Da and an octanol water partition coefficient logP should be less than five. The chrysophanol possess two hydrogen bond donor and four hydrogen bond acceptors. The logP value for chrysophanol is 2.810 [80]. Low lipophilicity and low logP values enhance the absorption or permeation of the drug. Conclusively, the chemical properties of chrysophanol recommends that it is suitable to be an oral active drug.

## 4. Biosynthesis

**Figure 1:**
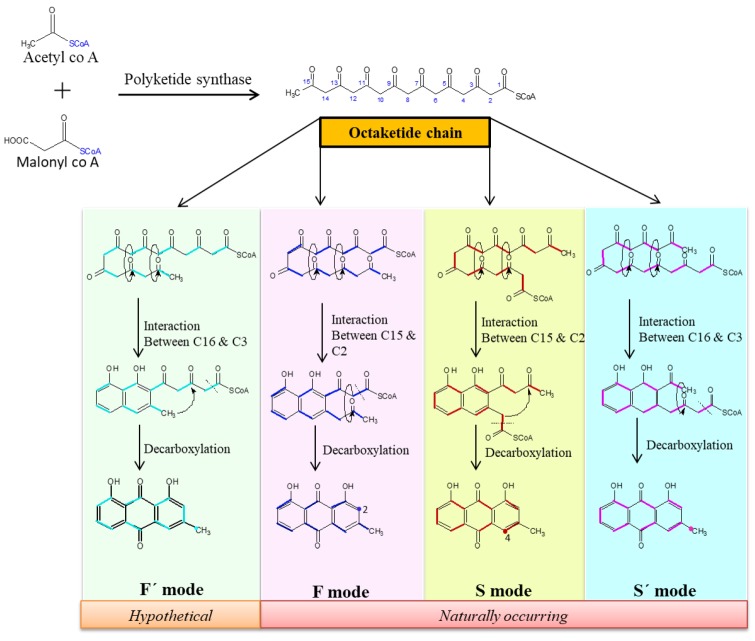
Different folding patterns of the octaketide chain. The bold lines indicate the carbon of the malonyl unit and bold point indicates the carbon of the acetyl unit. The curved arrow represents the aldol-type cyclization of reactions. Numbers 2 and 4, on the first ring of chrysophanol structure highlight the first carbon in the biosynthetic pathway.

Chrysophanol is naturally synthesized via the PMA or octaketide pathway; eight units of acetyl CoA are condensed by polyketide synthase. Leistner and Zenk [21] reported that chrysophanol is synthesized in fungi through the PMA pathway whereas in plants, it is synthesized through both shikimate and PMA pathways. The cyclization or folding pattern of the octaketide chain is different in different organisms. The occurrence of two folding patterns in nature is well established; these are categorized as “F” mode, named because of its occurrence in fungus and “S” mode, which is observed in the bacterial strain, *Streptomyces*. In plants, insects, and fungi, the polyketide chain is cyclized through “F” mode in which the first ring of polyketide is built by two C2 units (acetyl CoA), whereas in “S” mode, it is formed by three C2 units [22]. Bringmann and Irmer [81] designed a study to distinguish four possible folding patterns: the F and S modes, which were already known and the hypothetical S’ and F’ modes, which were never explored (Figure 1). Their study was accomplished by feeding ^13^C-labeled sodium acetate as the precursor of acetyl-CoA to different model organisms, such as *Nocardia* strain (bacterium), *Galeruca tanaceti* larvae (insect), callus culture of the torch lily, *Kniphofia uvaria* (plants), and *Drechslera catenaria* (fungi). Although, the authors did not succeed in ascertaining the existence of F’ mode, the possibility of S’ mode was proved through two-dimensional incredible natural abundance double quantum transfer nuclear magnetic resonance (2D INADEQUATE NMR) spectrum in which a new weak interaction, C3 to C4, was shown. This interaction might be due to the inter-acetate couplings, usually not found in the S mode. There is a further need to certify the F’ mode that is theoretically possible [81]. However, the F and S modes offer different intermediates but ultimately produce the same chemical structure through an aldol reaction, followed by decarboxylation. The first and middle rings of chrysophanol are synthesized via polyketide chain cyclization and the last ring, having methyl group, is formed through decarboxylation after aldol reaction within intermediate molecules. Emodin and emodinanthrone can form chrysophanol and chrysophanol anthrone, respectively, by dehydroxylation in the presence of the dehydrolase enzyme. Further oxidation of chrysophanol anthrone yields chrysophanol [82].

**Figure 2:**
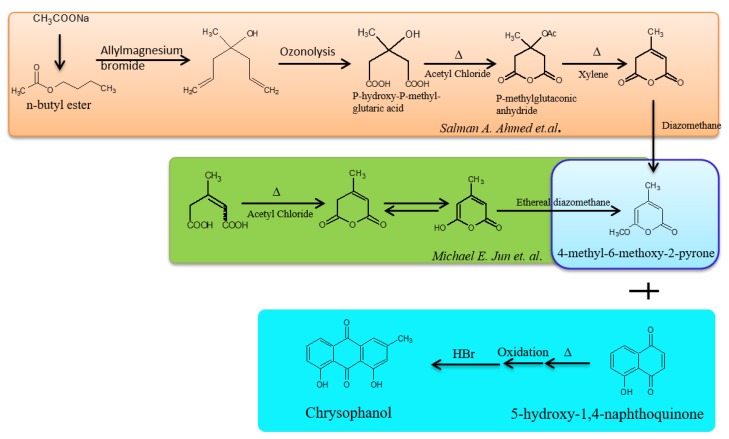
Chemical synthesis of chrysophanol.

Several researchers have attempted to chemically synthesize chrysophanol. Two methods using Friedel-Craft and Diels-Alder reactions, have been particularly studied; in both these reactions a common pyrone derivative, 4-methyl-6-methoxy-2-pyrone, is synthesized. This pyrone derivative when heated with juglone (5-hydroxy-1,4-naphthoquinone) and hydrolysed after oxidation, was shown to produce chrysophanol at a yield of 62% [83]. The synthesis of 4-methyl-6-methoxy-2-pyrone was found to differ in both the methods (Figure 2). The Diels-Alder reaction has an advantage over the Friedel-Crafts reaction in which pyrone might be converted into 2,6-dioxygenated pyrylium salt in the presence of strong electrophiles or Lewis acids. A convenient synthetic procedure of synthesizing pyrone was proposed in which the reaction was started with sodium acetate and ultimately 4-methyl-6-methoxy-2-pyrone was synthesized after seven steps and was finally converted to chrysophanol. However, all the methods of synthesis yield the same amount of chrysophanol (62% of all the reactants) and efforts to increase the yield have not been successful [76].

## 5. Pharmacology

For several decades, anthraquinones have been recognized as natural coloring and therapeutic agents. A range of pharmacological properties of anthraquinones has been reported, which include anti-oxidant, anti-ulcer, anti-inflammatory, anti-cancer, neuroprotective, anti-aging, lung protective, and hepatoprotective properties [84]. Structural relationship analysis of anthraquinones suggested that the methyl group on the 3rd position and two hydroxyl groups on the 1st and 8th position of chrysophanol are responsible for its anticancer effects [85]. Other biological activities might be due to the same. There is evidence that numerous TCMs are enriched with chrysophanol; this suggests its efficacy against various diseases. All the pharmacological activities of chrysophanol are listed in Table 2.

### 5.1. Anti-Diabetic Activity 

Chrysophanol exhibits antidiabetic activity through its participation in the insulin signaling cascade. It was reported to reduce postprandial hyperglycemia by 42.3% at a concentration of 0.18 mg/kg body weight in an albino mice model [100]. Arvindekar et al. [101] also reported that chrysophanol has potential to lower the blood glucose level up to 150 mg/dL as compared to the control at the dose of 2 mg/kg body weight after two hours of glucose feeding in male Wistar Albino rat. Although chrysophanol was found to be inactive against intestinal alpha glucosidase enzyme [101], it increased GLUT 4 (Glucose transporter type 4)-mediated glucose transport in differentiated L6 rat myotubules up to 100 μM concentration [102]. Chrysophanol also stimulated the phosphorylation of Insulin receptor substrate-1 (IRS-1) via an antagonistic effect on its inhibitor, PTP 1B (protein-tyrosine phosphatase 1B), a key target of the insulin signaling pathway. The authors demonstrated that chrysophanol competitively inhibited PTP 1B at an IC_50_ value of 12.3 µM, but could discriminate by not affecting highly similar structural homolog, tyrosine phosphatases, such as SHP1 (Src homology region 2 domain-containing phosphatase-1), SHP2 (Src homology region 2-containing protein tyrosine phosphatase 2), VHR (Vaccinia H1-Related), and TC-PTP (T-cell protein tyrosine phosphase). Furthermore, chrysophanol was reported to rapidly increase the level of P-AKT (phophorylated activated protein kinase B) at 50 µM in human liver carcinoma cells, Hep G2 [18]. P-AKT is a negative regulator of lipolysis, glyconeogenesis, and glycogen synthesis and a positive regulator of lipogenesis and protein synthesis. Therefore, chrysophanol is a worthy constituent of traditional medicine, Masiningan, which is used for diabetes mellitus. An *in-silico* study was performed to compare the efficacy of chrysophanol and a drug, Glibenclamide, against diabetes mellitus targets, namely β3adrenergic receptor (β3AR), Glycogen synthase kinase 3 (GSK3), PTP 1B, DPP4, PPAR-γ, Hexokinase, Potassium Voltage-Gated Channel Subfamily J Member 11 (KCNJ 11), Lipase, Alanine aminotransferase (ALT), Aspartate transaminase (AST), Glucose-6-phosphate dehydrogenase (G6PD), Fructose 1-6 bisphosphate (F6BPO_4_), Insulin receptors, Glutathione peroxidase (GPx), and Protein kinase C (PKC), using Glide tool. Chrysophanol docked well in the active site of DPP-IV (dipeptidyl peptidase 4) with a docking score of −5.67 and made five hydrogen bonds whereas Glibenclamide had a docking score of −3.9, with only three hydrogen bonds. Molecular dynamics studies suggested that chrysophanol could be used as a significant DPP-IV inhibitor [103]. Another *in-silico* study on the effect of chrysophanol with the peroxisome proliferator-activated receptor, PPAR-γ, using Glide tool, a molecular docking software revealed a Glide score of −3.92, which suggests that chrysophanol might act as an agonist of PPAR-γ, which is expressed in adipose tissue and regulates lipid and glucose metabolism [103]. These studies give directions for further studies employing in-vitro and in-vivo assays for certifying the agonistic effect of chrysophanol on PPAR-γ as well as on DPP-IV.

### 5.2. Anti-Cancer Activity

Several studies have demonstrated that chrysophanol exhibits anticancer properties mainly through necrosis, a caspase-independent phenomenon, which triggers an irreversible inflammatory response against tumor cells. Lu et al. [87] reported that chrysophanol reduced the cell viability in J5 human liver cancer cells in a concentration- and time-dependent manner through necrosis. However, apoptosis-associated signals, such as loss of mitochondrial membrane potential (MMP), increase in the number of reactive oxygen species (ROS) and cytosolic Ca^+2^, the release of cytochrome c from mitochondria, and delayed externalization of phosphatidyl serine in chrysophanol-treated J5 human liver cancer cells, allude to apoptosis. Annexin V/PI assay was performed to clarify this discrepancy and it was found that chrysophanol indeed induced necrosis because chrysophanol-treated cells were accumulated in the S phase and not in the sub-G1 phase (apoptotic population) of the cell cycle. Furthermore, the levels of apoptotic signal cascade associated proteins, such as Bax, apoptosis-inducing factor (AIF), Endo G, Apaf-1, Caspase-3, Caspase-8, Caspase-9, and Caspase-12, were found to decrease in the treated cells. Although, the level of cytochrome c was increased due to the chrysophanol treatment, the reduction in the ATP level and increase in lactate dehydrogenase (LDH) activity also pointed toward necrosis. The authors also validated the unregulated cell death process using inhibitors of the apoptotic pathway, z-VAD-fmk (pan-caspase inhibitor) and calcium ion chelator, BAPTA (1,2-bis(*o*-aminophenoxy) ethane-*N*,*N*,*N′*,*N′*-tetraacetic acid). No significant increase in cell viability was observed in both the cases [87]. Similarly, chrysophanol was shown to affect the cell viability in human renal cancer cells, Caki-2, and human lung cancer cells, A549 [88]. Reduction in the expression of cyclin D, cyclin dependent kinase 2 (cdk2), and thymidylate synthase in chrysophanol-treated A549 cells demonstrated cell cycle arrest at S phase in the necrotic population [86]. Cyclin D, cdk2, and thymidylate synthase are actively involved in DNA replication during the S phase of the cell cycle. A few decades ago, a receptor-interacting serine/threonine-protein kinase 1 (RIP1) was disclosed as a central player in the necrosis cascade, which acts as a negative regulator of apoptosis via activation of NF-κB. The inhibition of RIP1 was observed in chrysophanol-treated Caki-2 human renal cells at 20 μM, whereas significant inhibition of phosphorylated STAT3, a transcription factor of cell viability and negative regulator of apoptotic cascade and stress-induced signal transducing kinases, c-Jun N-terminal kinase (JNK), and extracellular signal-regulated kinase (ERK) were detected in immunoblot analysis at 2 μM [88]. Cytotoxicity of chrysophanol was also observed against human breast cancer cell lines, MCF-7 and MDA-MB-231, and against human leukemia cells, HL 60 and L1210 [29,104]. Chrysophanol-treated HL60 cells did not display apoptosis induction [104]. However, no mode of action was determined in both the human breast cancer cell lines. The cdk25 B phosphate, a crucial enzyme in cell cycle regulation removes phosphate from the active site of CDK and is involved in G2-M and S phase progression. Chrysophanol could inhibit Cdc25B phosphatase with an IC_50_ value of 10.7 μg/mL, indicating its potential as a therapeutic agent for cancer chemotherapy [105].

Chrysophanol was observed to block the epidermal growth factor receptor (EGFR)/mammalian target of rapamycin (mTOR) signaling pathway in SNU-C5 human colon cells. The EGFRs are overexpressed in colon cancer cells and its activation through epidermal growth factor (EGF) is responsible for the proliferation, survival, and metastasis of cells. Chrysophanol suppressed the transformation of EGFR to phosphorylated EGFR, which resulted in the regression of its downstream signaling molecules, such as AKT, ERK, and the mammalian target of rapamycin (mTOR)/ribosomal protein S6 kinase (p70S6K) [89]. The cytotoxic efficiency of chrysophanol was also investigated using two chorio carcinoma cells, JAR and JEG3; the viability of JEG3 but not of JAR cells was found to be diminished by the induction of apoptosis [56]. In another study, chrysophanol showed anticancer activity against human tumor cell lines, A549 (non-small cell lung), SK-OV-3 (ovary), SK- MEL-2 (melanoma), XF498 (central nervous system), and HCT-15 (colon) with IC_50_ values of 24.76, 7.28, 5.83, 30.0, and 30.0 μg/mL, respectively [106]. Chrysophanol competively inhibited cathepsin B with an IC_50_ value of 0.7 μM. Cathepsin B was found to be overexpressed in tumour cells, and was mainly involved in the metastasis and invasion of tumour cells [107]. The role of chrysophanol was also investigated in a protein–ligand interaction analysis using AutoDock Vina 4.0, with casein kinase II subunit A (CK II A) and human myosin light chain kinase (MYLK) member 4 (MYLK4); CK II A regulates numerous cellular processes, such as cell cycle progression, apoptosis, and transcription, and MYLK 4 has a specific effect on lung and breast cancers [85]. The results suggested that chrysophanol is a potent agonist of CK II A and is antagonistic to MYLK4. However, the effect of chrysophanol on metastasis and invasion of tumour cells remains unexplored.

### 5.3. Neuroprotective Effects

The protective effect of chrysophanol on lipopolysaccharide (LPS)-induced inflammatory and oxidative stress response of BV2 murine microglial cells was investigated [95]. Chrysophanol was found to modulate the MAPK signaling, resulting in the downregulation of ERK, p38, and JNK, as well as of nitric oxide synthase (NOS) and cyclooxygenase (COX) along with the products of both the enzymes, nitric oxide (NO) and prostaglandin (PGE2). The pro-inflammatory cytokines, TNF-α, interleukin 1 (IL-1), and IL-6 were also suppressed in the microglial cells. The activation of microglial cells (BV2 cells) and the release of pro-inflammatory cytokines are the main characteristics of neurodegenerative diseases, including Parkinson’s (PD), Alzheimer’s (AD), multiple sclerosis (MS), AIDS dementia complex, cerebral stroke, and traumatic brain injury. The administration of chrysophanol significantly reduced the intracellular ROS-mediated cell damage and inhibited DNA oxidation in BV2 cells through positive regulation of antioxidant enzymes, such as superoxide dismutase (SOD), and glutathione (GSH) in BV2 cells, which eventually reduced the oxidative stress in BV2 cells. Chrysophanol induced the anti-apoptotic factor, BCL-2, and inhibited the pro-apoptotic factors, BAX and AIF, together with ROS and mitochondrial fission by attenuating the phosphorylation of dynamin-related protein 1 (Drp1) in the glutamate induced hippocampal neuronal cells, HT-24 [96]. Hippocampus, which is mainly affected by Alzhiemer’s disease (AD), acts as the major region that controls the short- and long-term memory. Moreover, the effect of chrysophanol on hippocampus of lead-poisoned neonatal mice was examined, wherein it was observed that chrysophanol lessened the hippocampal injury and enhanced the learning memory by inducing the antioxidant defence system of the cells. The lead content in blood, brain, heart, spleen, liver, and kidney in the lead-exposed neonatal mice was also found to be reduced in a dose-dependent manner [97]. Chrysophanol could control the depressive behaviour in LPS-induced ICR mice when it was administered intraperitoneally, once daily, for seven consecutive days at a concentration of 20 mg/kg body weight. The protein expression analysis using hippocampus serum of LPS-induced ICR mice was performed using western blotting to understand the possible target pathway. The inhibition of LPS-stimulated cytokines, IL-6, IL-1β, and TNF-α, along with that of P2X7 (purinoceptor 7), p-IKKα (IκB kinase α), p-IKKβ (phospho- IκB kinase β), p-IkBα (phospho-inhibitory subunit of NF-KBα), and p-NF-kBp65 (Phospho-NF-κB p65) suggested that the anti-depressive effect of chrysophanol was due to the modulation of P2X7/NF-κB pathway [97]. Chrysophanol can, thus, be a potent therapeutic agent for AD. Numerous studies have reported that chrysophanol could efficiently alleviate cerebral ischemia/reperfusion (I/R) in mice. The efficacy of chrysophanol containing liposomes has been explored against middle cerebral artery occlusion (MACO) injured mice and it has been observed that chrysophanol proficiently reduced the oxidative stress by inducing the antioxidant enzymes, SOD and GSH, and by downregulating the apoptosis associated factors, such as Bax, caspase-3, and cytochrome c [108]. NACHT, LRR and PYD domains-containing protein 3 (NALP3) inflammasome, which is activated through the signals of tissue injury, was found to be suppressed along with its downstream factors, caspase-1 and IL-1β, in chrysophanol-treated transient middle cerebral artery occlusion (tMCAO) in male CD1 mice when injected with chrysophanol [97]. This proved that chrysophanol confers long term neuroprotection against ischemic brain injury in a murine model of focal cerebral I/R (ischemia/reperfusion) by halting the signaling cascade of NF-κB and P50/P65. Recently, the possible mechanism of action of chrysophanol on cerebral ischemic stroke caused by endoplasmic reticulum stress was elucidated [98]. This study demonstrated the downregulation of transcription factors mainly involved in ischemic brain namely, GRP78, p-eIF2a (phospho-elongation initiation factor 2a), CHOP (CCAAT-enhancer-binding protein homologous protein), and caspase-12, in chrysophanol-treated ischemic brain and upregulation of IκB-a level, an inhibitor of NF-κB. Apart from enhancing the learning activity, chrysophanol has the potential to lessen retinitis pigmentosa (RP), an inherited photoreceptor-degenerative disease. It inhibited apopotosis, gliosis, activation of micro-glia, and matrix metallopeptidase 9 (MMP-9) expression in *N*-methyl-*N*-nitrosourea (MNU)-induced mouse model of RP [109].

### 5.4. Hepatoprotective Activity

Chrysophanol has been reported to possess hepatoprotective activity. It has been shown to block the RIP 140/348 and NF-κB pathway, and to regulate the inflammatory response to cells in LPS/D-GalN-induced acute hepatic injury in mice resulting in the reverse regulation of TNF-α, IL-6, and NOS. The inhibition of caspases associated with apoptosis induction indicated that chrysophanol could protect against liver injury through its anti-apoptotic activity. The oxidative stress-related factors were also observed to be altered to enhance the cell viability [90]. The hepatoprotective activity of chrysophanol was studied in ethanol-induced HepG2/CYP2E1 cells at a concentration of 100 µM and it was found to significantly reduce the gamma-glutamyl transpeptidase (GGT) activity, which is required for GSH homeostatsis [65]. In addition, immunoblot analyses revealed a reduction in the expression of GGT, GSH, and cytochrome P-450 2E1 (CYP2E1). This study suggested that chrysophanol could be a potential candidate for offsetting ethanol-induced liver injury.

### 5.5. Anti-Ulcer Activity

A recent study suggested that chrysophanol and its rich extract of medicinal plants significantly protects against gastrointestinal effects of cold-resistant ulcer, alcohol, aspirin, and pyloric ligation-induced ulcer in rats [110]. Chrysophanol efficiently reduced the total and free acids by inhibiting the H^+^/K^+^-ATPase activity in-vitro at IC_50_ value of 187.13 µg/mL. However, it exhibited less activity than emodin. Upregulation of mucin secretion, a defensive mechanism of ulcer was also observed in chrysophanol treated mice.

### 5.6. Anti-Inflammatory Activity

Anti-inflammatory properties of chrysophanol have been explored by many researchers to define the protective action mechanism in various diseases [76]. Kim et al. [91] studied the effects of chrysophanol on dextran sulfate sodium (DSS)-induced colitis and LPS-induced inflammatory responses in mouse peritoneal macrophages. Chrysophanol was reported to inhibit the production of TNF-α and IL-6, and the expression of COX-2 upon treatment with LPS. It proficiently suppressed the activation of NF-κB and caspase-1 in LPS-stimulated macrophages. In addition, the efficacy of chrysophanol against phorbol 12-myristate 13-acetate and calcium ionophore A23187 (PMACI)-treated human mast cells HMC-1 was investigated. The intracellular calcium levels and histamine release were inhibited, as was also observed for proinflammatory cytokines, IL-1β, IL-6, TNF-α, and thymic stromal lymphopoietin (TSLP). In addition, there was an increase in the levels of phosphorylated-mitogen-activated protein kinase in PMACI-treated HMC-1 cells that were pre-treated with chrysophanol. It has also been suggested that chrysophanol significantly eradicates atopic dermatitis. It is an active component of AST2017-01, which is a novel, potentially anti-inflammatory, KHM (konsentrasi hambat minimum) or functional food [111]. Anti-inflammatory of chrysophanol containing aqueous extract of *R. patientia* is also examined using carrageenan, histamine, dextrane, serotonine formaldehyde-induced oedema tests, cotton-pellet granuloma, and Kabak tests in rats [112].

### 5.7. Anti-Viral Activity

The antiviral activity of chrysophanol against poliovirus types 2 and 3, Coxsackievirus types A21 and B4, human rhinovirus type 2 (*Picornaviridae*), and the enveloped viruses, Ross River virus (*Togaviridae*) and herpes simplex virus type 1 (*Herpesviridae*), has been investigated using in-vitro assays [57]. It could significantly inhibit the replication of poliovirus type 2 and 3. It also exhibited an anti-cytopathogenic effect on poliovirus types 2 and 3 in BGM (Buffalo green monkey) kidney cells at EC_50_ values of 210 and 20 µg/mL, respectively, and an irreversible virucidal effect was observed on poliovirus particles. Chysophanol attenuates viral replication at an early stage. Because chrysophanol is structurally similar to other anthraquinones, emodin, aloe-emodin, rhein, and 1-8 dihydroanthraquinone, which are not active against poliovirus, it is suggested that the methyl group attached to C-3 position is responsible for the antiviral effect of chrysophanol. The effect of chrysophanol against Japanese encephalitis virus (JEV) was demonstrated through plaque reduction and virucidal activity assays; the IC_50_ values were 15.82 and 0.75 µg/mL, respectively. Chrysophanol inhibited 90% of the JEV yield at 10 μg/mL. It also triggered host innate immune response against JEV infection by inducing the activity of the gamma interferon activation site (GAS), which is normally activated by IFN-γ [113]. The authors reported that chrysophanol did not have any effect against vesicular stomatitis virus, herpes simplex virus types 1 and 2, parainfluenza virus, and vaccinia virus at 50 µg/mL [113]. In addition, chrysophanol has moderate efficiency in inhibiting HIV-1 protease, which is an essential enzyme involved in the life cycle of human immunodeficiency virus [114].

### 5.8. Anti-Fungal Activity

Chrysophanol exhibited fungicidal effects against *Blumeria graminis* f. sp. *hordei*, the causative agent of barley powdery mildew, with an IC_50_ of 4.7 μg/mL. It also showed a protective effect against cucumber powdery mildew caused by *Podosphaera xanthii* at 100 μg/mL [115]. The protective effect was due to the breakdown of the cell wall of germ tubes, swelling up and the collapse of hyphal tips, hyphal malformation, delayed and reduced sporulation of fungus and spore germination, and appressorial formation and penetration. The morphological changes, such as haustorium deformation, vacuolization, abortion, and necrosis were detected in chrysophanol-treated fungus strain [116]. These studies demonstrate that chrysophanol can be a beneficial agent for crop protection. Chrysophanol was found active against *Candida albicans*, *Cryptococcus neoformans*, *Trichophyton mentagrophytes*, and *Aspergillus fumigatus* with minimum inhibitory concentration values (MIC) values of 50, 50, 25 and 50 μg/mL, respectively [76]. The antifungal property of chrysophanol was first observed in 1877 against the ring worm [76]. It was also tested against *Botrytis cinerea* and *Rhizoctonia solani* at a concentration of 500 μg/mL. The percent inhibition for both these pathogens was 21.2 and 22.5%, respectively [117].

### 5.9. Anti-Bacterial Activity

For many decades, chrysophanol has been explored as a promising antibacterial agent against some human pathogens [118]. However, contradictory results have been documented for the minimum inhibitory concentration (MIC) values against *Escherichia coli* [76]. Therefore, further studies are needed to validate the findings. The previously reported MIC values against different strains are listed in Table 3. The biofilm architecture of bacterium serves as a protective layer for the penetration of antibiotics [118,119]. Recently, the antibiofilm activity of chrysophanol (200 μM) against *Pseudomonas aeruginosa* and *Stenotrophomonas maltophilia* was reported [120]. Although many virulence factors and drug efflux pumps of bacteria participate in the defense mechanism, the effect of chrysophanol on these has not been examined.

### 5.10. Miscellaneous Activities

Several other pharmacological studies on chrysophanol have been documented. It is considered as a moderate antiprotozoal agent against chloroquine resistant (W2) and sensitive (D6) strains of *Plasmodium falciparum* [47]. Chrysophanol manifested anti-obesity response in macrophyage cell line RAW264 and 3T3-L1 adipocytes by modulating expression of the proinflammatory cytokines level IL-6, TNF-α, MCP-1 of NF-κB pathway and adiponectin production, an anti-inflammatory molecule secreted by adipose tissue. It could also recover pulmonary injury in mice through strong anti-inflammatory and antioxidant retort to injured tissue [93]. Chrysophanol significantly inhibits cholesterol and triglyceride in zebra fish provided with high fat/cholesterol diet. It might be attributed to the promotive effect on digestion and decreased absorption of dietary lipid; however, the exact mechanism of hypolipidemic activity of chrysophanol is unexplored as of yet. The hypolipidemic property of chrysophanol offers a clue to researchers to discover a new drug for lipid metabolic disorders. Chrysophanol possesses anti-tuberculosis activity with an IC_50_ value of 64 µg/mL against *Mycobacterium tuberculosis* H37Ra and *M. bovis* [123]. Furthermore, an *in-silico* study was performed in order to understand the mode of action; the interaction of chrysophanol with the enzyme TDP-6-deoxy-D-xylo-4-hexulose 3, 5-epimerase (RmlC), which is involved in cell wall synthesis of *M*. *tuberculosis* was computed using Autodock tool. The obtained docking score of −9.24 kcal/mole proved that it is a valid mode of action [76]. The antinemic activity of chrysophanol against *Meloidogyne incognita J2s* (root-knot nematode) was also reported with an ED_50_ value of 102.59 mg/L [124]. Moreover, chrysophanol glycosides are a major component of plants like cassia, and aloe exhibited the laxative effect [76]. The mechanism behind the laxative effect is considered as the glycosidic bond hydrolysed by intestinal microbes and aglycone form “chrysophanol” is absorbed by intestinal epithelial cells and enhances the mucous secretion stimulation; eventually an increase in intestinal water content gets relief from constipation. Chrysophanol possesses the C-1 and C-8 hydroxyl groups which are thought to play a vital role in the laxative effect [84]. The amount of total anthraquinone glycosides is used as a quality marker of the laxative drug. The ASEAN herbal medicine recommended it should not be less than 0.5 % of dried leaf raw materials to be a laxative drug [84]. The chrysophanol was also identified as a deer repellent compound in sickle pods weeds [123]. Chrysophanol appeared to be a better angiotensin enzyme inhibitor than captopril when analysed using the AutoDock Vina software. The angiotensin inhibitors are considered as good chemotherapeutics agents for hypertension and cardiovascular diseases [85].

## 6. Pharmacokinetics

Numerous pre-clinical pharmacokinetic studies revealed that chrysophanol exhibited better absorption and slower elimination at higher concentrations than some other anthraquinones obtained from the Rhubarb family. Chen et al. [92] studied the plasma protein binding (PPB) rate in rat plasma, human plasma, and bovine serum albumin and reported values of 83 ± 2%, 88 ± 3%, and 58 ± 3%, respectively. The PPB rate is an important parameter in pharmacokinetics and pharmacodynamics that strongly influences the bioavailability, metabolism, and tissue distribution of a drug. The high PPB rate of chrysophanol could be responsible for its tissue distribution in the body. The levels of chrysophanol were found to be higher in kidneys than in liver, suggesting that it is eliminated by excretion rather than by being metabolised. However, chrysophanol cannot easily cross the blood–brain barrier, resulting in low levels of chrysophanol in the brain tissue. Thus, increasing the hydrophilicity of chrysophanol is desired considering its potential to alleviate neurodegenerative diseases, such as cerebral ischemia-reperfusion [92]. Quyu Qingre granules (QYQRGs), a TCM used for treating blood stasis syndrome, which possesses two major anthraquinones, chrysophanol and rhein, has been evaluated for its efficacy in normal and acute blood stasis model rabbits. Higher AUC_(0–∞)_ and C_max_, and lower T_max_ and clearance rate of chrysophanol were obtained in the group of rabbits affected with acute blood stasis than in the normal rabbits after oral administration at a dose of 2.0 g/kg b.w. The data for QYQRG suggested that acute blood stasis syndrome enhanced chrysophanol absorption and reduced its elimination but the distribution of chrysophanol was less in blood stasis syndrome [11]. In another study, chrysophanol was used as a marker to evaluate the pharmacokinetics of TCM, Dahuang Fuzi Decoction (DFD), and Radix et Rhizoma Rhei. An increase in C_max_ and T_max_ and a decrease in AUC_(0–t)_ and AUC_(0–0)_ in the group administered with DFD indicates the composition of decoction could be able to modulate the action of cytochrome P450, the major phase I drug metabolizing enzyme and drug effux pump, resulting in slower absorption [125]. The bioavailability of chrysophanol has been investigated using human colon adenocarcinoma cancer cells line, Caco-2, wherein it was found that the intracellular accumulation of chrysophanol was 414.02 nmol/L/mg protein after 10 min of administration and no saturation stage was observed until the concentration of 200 µM. Furthermore, the inhibition of p-glycoprotein efflux pump increased the absorption of chrysophanol [126]. Sreelakshmi et al. [80] predict the ADME (absorption, distribution, metabolism and excreation) values by the *in-silico* approach. The ADME values of chrysophanol were −3.5 for aqueous solubility, 3 for aqueous solubility level, 0 for human intestinal absorption level (HIA) and 2 for blood brain barrier (BBB) penetration level. These values were matched with standard ADME prediction chart and found that good aqueous solubility, desirable intestinal absorption and medium or low blood brain barrier penetration ability. These predictive values were strongly correlated with in vivo studies.

## 7. Toxicology

Apart from enormous pharmacological studies, researchers carried out experiments to understand the toxicity of chrysophanol. The Ames test was performed to elucidate its mutagenicity. It was found that it exhibited strong mutagenicity on two *Salmonella* strains, TA 2637 and TA 1537, with metabolic activation or without metabolic activation. No mutagenic effect was observed in two strains, TA98 and TA100 [127]. In addition, comet assay, micronuclei induction, and mutation induction tests were employed using mouse lymphoma cells, L5178Y, at various concentrations (30–100 μM). It was found that the relative mutation frequency in chrysophanol-treated mouse lymphoma cells was not exceeded more than two-fold compared to the control. The number of the micronucleated cells was not found to increase in a concentration-dependent manner and in comet assay, no significant changes were observed in the genetic material of chrysophanol-treated cells; chrysophanol, thus, imparts moderate toxicity [128]. Furthermore, the resonance light scattering technique was implemented to evaluate the interaction of chrysophanol with DNA and its efficiency to exert a toxic effect through the saturation value binding DNA [129]. This study concluded that chrysophanol binds to DNA in a similar manner as ethidium bromide, mitoxanthrone, adriamycin, but it is not potentially toxic as the others are; the saturation value of drug binding for chrysophanol was 0.53 whereas it was 3.31, 10.58, and 14.70 for mitoxanthrone, adriamycin, and ethidium bromide, respectively. Mengs et al. [130] investigated the chromosomal aberration potential of chrysophanol in Chinese hamster ovary (CHO), and concluded that it had no clastogenic potential up to its solubility limit. The mutagenic effect of chrysophanol on other mammalian cells, rat hepatocytes, and v79 was also investigated in the unscheduled DNA synthesis test and hypoxanthine-guanine phosphoribosyl transferase test. The results of both the assays revealed negligible toxic effects of chrysophanol. The oral toxicity of chrsyphanol for rat model and ocular irritancy has been predicted by LD_50_ module and ocular irritancy module of the TOPKAT (toxicity prediction by computer assisted technology) package. The obtained LD_50_ value for chrysophanol was 2.5 g/kg. Higher LD_50_ values means the compound is toxic at very high dose. The obtained values for ocular irritancy represented that it does not show irritancy in two models but little irritancy was shown in the third model [79]. However, the in vivo model has not been used to explore the toxicity of chrysophanol as of yet.

## 8. Recent Advances

In recent times, researchers have used various theoretical and practical concepts to improve the understanding of pharmacology and pharmacokinetics of natural compounds. Lee et al. [131] designed experiments to improve the chrysophanol content in adventitious roots of *Aloe vera* and found that salicylic acid modulates the expression of octaketide synthase gene. They treated the *A. vera* adventitious roots cultured on liquid Murashige and Skoog’s medium with 0.3 mg/L indolebutyric acid for 35 days with plant-derived elicitors such as salicylic acid, methyl jasmonate, and ethephon. It was found that the content of chrysophanol was significantly increased by 5 to 13 times in the salicylic acid-treated group of *A. vera* adventitious roots compared to that in the untreated control. The application of nanotechnology using the green chemistry approach has led to novel pharmacokinetic properties of natural entities. Several hydrophobic phytomolecules, mainly phenolics and alkaloids, have been functionalised either with nanometal or nano-organic molecules to improve their solubility and bioavailability. Nanomedicines deliver a drug systematically into blood plasma and ultimately improve its bioavailability. Gold chrysophanol decorated with poly (DL-lactide-co-glycolide) nanoparticle was synthesized and tested against prostate cancer. Although free chrysophanol commonly leads to cell death through necrosis, its nanoparticles induced apoptosis in LNCap human prostate cancer cells. The increased expression of CHK1 and p27, and a decrease in the expression of CDK1 and cyclin D1 certified the arrest in the cell cycle in sub-G phase. The nanoparticles of chrysophanol reversed the expression of histone deacetylase, HDAC6, which regulates the cell shape and migration. The nanoparticles were observed to target p53/ROS crosstalk to prevent proliferation. Chrysophanol was able to reduce the tumor volume and weight in mice. Pharmacokinetic studies in mice revealed that injection of chrysophanol nanoparticles exhibited high bioavailability compared to free chrysophanol [132].

For a few decades, researchers have been engaged in establishing the tissue culture of medicinal plants so as to obtain their bioactive molecules in large scale and at low cost without harvesting the natural resources. Owing to the ability of endophytes to produce similar compounds as their hosts, the co-culture technique with host plants and their endophytes offers the opportunity to enhance the accumulation of bioactive compound in host plants. Ding et al. [133] designed a study in which seedlings of *R. gmelini* Turcz (RGT) were co-cultured with its isolated endophytes, *Aspergillus* species, *Fusarium* species, and *Ramularia* species. under optimized conditions. The results showed that the chrysophanol content was increased by 3.60-fold in the co-culture of RGT and *Aspergillus* spores compared to that in control [133]. Zhao et al. [134] studied the photo-physicochemical behavior of chrysophanol and assessed the time-dependent density functional theory (TD-DFT) to obtain meaningful information about the absorption spectrum, lowest triplet excited-state energy, vertical electron affinity, and vertical ionization potential. They proposed that chrysophanol could be a photodynamic medicine for clinical therapy of the diseases occurring on the shallow surface and for vascular capillary diseases [134].

## 9. Conclusions and Future Prospects

Chrysophanol is a unique compound which is cosmopolitan in distribution and is synthesized in an organism-specific manner. Because of its presence in several TCMs, it has been studied extensively and its multifarious pharmacological activities, which are anti-diabetic, anti-inflammatory, anti-cancer, anti-ulcer, anti-microbial, neuroprotective, and hepatoprotective, have been reported. The mechanisms of action of chrysophanol against several diseases have been considerably explored; it strongly modulates the NF-κB, EGF/mTOR, and MAPK pathways. The effect of chrysophanol has not been reported against lipid metabolic disorders; however, its lipid lowering activity has been documented. Pharmacokinetics studies suggest that the rate of its absorption is higher than that for other anthraquinones and its tissue distribution is in the order kidney > liver > heart > brain. The mobility of chrysophanol across the blood–brain barrier is poor, which is a hindrance, considering the fact that it is a good neuroprotective agent. The nanotechnology approach has been implemented to enhance the bioavailability and to reduce the toxicity associated with chrysophanol by producing chrysophanol liposomes and chrysophanol-coated gold nanoparticles. Although the potential therapeutic value of chrysophanol is high, it is not considered as a single-therapy drug because of its toxicity, which is possible to be controlled in formulations like in TCM and TKM. However, chrysophanol was not found too much toxic in in-vitro assays; it has, however, not been subjected to in vivo toxicity studies, to date. The moderate toxicity observed for chrysophanol can be used for anti-genotoxic effect. Besides its pharmacological activity, chrysophanol is also proposed as a coloring agent for use as a food additive. Further studies are needed to utilize the complete spectrum of activities shown by chrysophanol.

## Figures and Tables

**Table 1:** Natural sources of chrysophanol.

Family	Genus	Species	Plant Part		References
**Plants**					
Polygonaceae	Rheum	*R. emodi*	Root and rhizome		[24,29]
		*R. palmatum*	Root		
		*R. undulatum*	Root		
		*R. officinale*	Root		
		*R. rhabarbarum*	Root		
		*R. nobile*	Rhizome		[30]
	Rumex	*R. crispus*	Root		[24]
		*R. dentatus*			
		*R. acetosa*			
		*R. obtusifolius*			
		*R. hymenosepalus*			[24]
	Polygonum	*P. multiflorum*	Root		[24]
		*P. cillinerve*	Rhizome		[31]
Rhamnaceae	Rhamnus	*R. alpinus*	Bark		[32]
		*R. saxatilis*			
		*R. frangula*			[33]
		*R. purshiana*			[34]
	Berchemia	*B. floribunda*	Root		[35]
	Colubrina	*C. greggii*	Root		[36]
Fabaceae	Cassia	*C. tora*	Seeds, stem		[7,8]
		*C. alata*	Leaves		
		*C. fistula*	Seed, wood, leaves, Seeds, roots and bark		
		*C. absus*	Root		
		*C. acutifolia*	Root, leaves		
		*C. angustifolia*	Leaves, pods, callus cultures from coteledons		
		*C. auriculata*	Pod husk		
		*C. biflora*	Flower		
		*C. didymobotrya*	Leaves, pods, root bark		
		*C. barrettiana*	Heartwood		
		*C. glauca*	Stems		
		*C. grandis*	Seeds		
		*C. italica*	Leaves, pods		
		*C. javanica*	Leaves, seeds		
		*C. laevigata*	Seeds, pods		
		*C. marginata*	Seeds, leaves, wood		
		*C. mimosoides*	Aerial part		
		*C. nomame*	Aerial part		
		*C. obtusifolia*	Roots, seeds		
		*C. occidentalis*	Leaves, seeds		
		*C. podocarpa*	Leaves, callus culture		
		*C. pudibunda*	Roots		
		*C. pumila*	Whole plant		
		*C. racemose*	Stem bark		
		*C. renigera*	Leaves		
		*C. reticulta*	Leaves		
		*C. siamea*	Leaves, stem, bark root bark		
		*C. sophera*	Flower root bark stem bark		
		*C. spectabilis*	Leaves, flower, buds		
	Senna	*S. italica*	Pods		[37]
		*S. macranthera*	Bark		[38]
	Sophera	*S. flavescens*	Root		[39]
Liliaceae	Simethis	*S. bicolor Kunth*	Root		[40]
	Eremurus	*E. anisopterus*	Aerial part		[41]
		*E. spectabilis*	Leaves		[42]
		*E. chinensis*	Whole plant		[41]
Asphodelaceae	Aloe	*excelsa*	Leaves		[43]
		*A. vera*	Leaves		[44]
		*A. ferox*	Leaves		[45]
		*A. grandidentata*	Leaves		[46]
		*A. pulcherrima*	Root		[47]
		*A. barbadensis*	Leaves		[48]
		*A. hijazensis*	Root		[49]
	Bulbine	*B. narcissifolia*	Leaves		[50]
		*B. natalensis*	Leaves, root		[51]
		*B. abyssinica*	Roots, fruits		[52]
	Kniphofia	*K. isoetifolia*	Root		[53]
	Asphodelus	*A. tenuifolius*	Whole plant		[54]
Buphorbiaceae	Cluytia	*C. hirsuta*	Leaves		[55]
Hemerocallidaceae	Dianella	*D. longifolia*	Root		[56]
Meliaceae	Walsura	*W. trifoliata*	Bark		[57]
Picramniaceae	Alvaradoa	*A. amorphoides*	Stem bark		[58]
Podocarpaceae	Podocarpus	*P. fleuryi*	Twigs		[59]
Dipterocarpaceae	Shorea	*S. worthingtonii*	Timber		[60]
**Fungus**					
Hypocreaceae	*Trichoderma*	*T. viride*		Sugarcane Endophyte	[61]
		*T. polysporum*		Root endophyte	[28]
		*T. aureoviride* PSU-F95		Marine	[62]
		*T. harzianum,*		Lab isolate	[27]
Pleosporaceae	*Curvularia*	*C. lunata*		Marine sponge	[26]
	*pachybasium,*	*P. candidum,*		Endophyte	[63]
Didymellaceae	*Phoma*	*P. exigua, P. foveata*		Pathogen	[64]
Trichocomaceae	*Aspergillus*	*Aspergillus* species		Marine fungus	[65]
	*Penicillium*	*P. citrinum* SCSGAF 0167		Mangrove and marine fungus	[26]
		*P. islandicum* Sopp		Icelandic cultured dairy product	[66]
		*P. citrinum* PSU-F51		Marine oraganism	[26]
		*P. oxalicum* 2-HL-M-6		Mangrove sed.	[67]
		*P. oxalicum*		Curcuma wenyujin	[68]
	*Paecilomyces*	*P*. species (Tree 1–7)		Bark of a mangroove	[69]
Dothideomycetes	*Monodictys*	*M*. species		Marine Organism endophytes	[70]
Pleosporaceae	*Drechslera*	*D. holmii* and *D. ravenelii*		Endophytes	[71]
		*D. catenaria*			
	*Phaeospheria*	*P. spartinae RKDO785 and RKDO808*		Marine isolates sea foams	[72]
Montagnulaceae	*Paraconiothyrium*	*P. brasiliense*		Endophyte	[73]
**Lichen**					
Parmeliaceae	*Asahinea*	*A. chrysantha*		Whole thallus	[74]
**Insect**					
Chrysomelidae	*Galeruca*	*G. tanaceti*		Eggs	[75]
	*Galerucella*	*G. tenella*		Adults	[76]
		*G. pusilla,*			
		*G. calmariensis*			
		*G. lineola*			
	*Trirhabda*	*T. geminata*		Larvae	[76]
Adelgidae	*Adelges*	*A. tsugae*		Eggs, adult	[76]
**Bacteria**					
Streptomycetaceae	*Streptomyces*	*S*. species		Terrestrial	[76]

**Table 2:** Pharmacological activities of chrysophanol.

Biological Activity	Study Model	Mode of Action	Target	Effective Concentration	Reference
Anticancer	Human lung cancer A549	↑ Reactive oxygen species (ROS) and Ca^+2^ ion ↓ Mitochondria membrane potential and adenosine triphosphate Trigger DNA damage Induce S phase cell cycle arrest	Necrosis	50 μM	[86]
	J5 Human liver cancer cell line	↑ ROS and Ca^+2^ ↓ Mitochondrial membrane potential and ATP, ↑ LDH, ↓ AIF, Endo G, Apaf-1, Caspase-3, Caspase-8, Caspase-9 and Caspase-12 ↑ Cytochrome c, Bax, SOD (Cu/Zn), SOD (Mn), catalase and GST	Necrosis	120 μM	[87]
	Human renal cell carcinoma Caki-2 cell	↑ ROS RIP1, STAT3-P	Necrosis	20 μM	[88]
	SNU-C5 human colon cancer cell	↓ P-EGFR, P-AKT, P-MTOR	EGFR/MTOR signaling	120 μM	[89]
Hepatoprotective	LPS)/D-galactosamine (GalN)-challenged acute liver injury in mice	↓ Ratio of Bax/Bcl-2, caspase-3 and caspase-8 ↓ TNF-α, IL-6 ↑ IL-10 content Restore the SOD, GPX, GSH, CAT	RIP 140/348 and NF-κB pathway	1, 10 mg/kg	[90]
Anti-inflammatory	Peritoneal Macrophage Culture of Male C57BL/6 and female BALB/c mice,	↓ LPS-induced NF- κB activation ↑ Caspase-1 activation Proinflammatory cytokine production (TNF-α, IL-6, COX-2, and iNOS)	NF-κB pathway	2 and 20 µM	[91]
Antiobesity	Macrophage cell line RAW264 and 3T3-L1 adipocytes	↑ Adiponectin cytokine, ↓ IL-6, TNF α, MLP-1	NF-κB pathway	10 and 100 µM	[91]
Hypolipidemic	Danio rerio	↓ cholesterol and triglyceride	Need to explore	0.6 µM and 6.4 µM	[92]
Pulmonary injury	BALB/C mice	↑ PPAR-γ ↓ IL-6, TNF-α	NF-κB pathway	10 and 20 mg/kg/day	[93]
Antidiabetic	L6 rat myoblasts (CRL-1458TM)	GLUT 4 mediated glucose transport ↓ PTP 1 B	Insulin signalling cascade	100 µM	[94]
	HEP G2 cells	PTP1B, ↑ P-AKT ↓ lipolysis, glyconeogenesis and glycogen synthesis	Insulin signalling cascade	12.3 µM	[84,87]
	*In-silico*	PPAR-γ agonist	Insulin signalling cascade	GLIDE SCORE −3.92	[84]
Neuroprotective	BV2 murine microgial cell	ERK, P-38 and JNK, nitric oxide synthase and cyclooxygenase, and prostaglandins PGE2. ↓ Proinflammatory cytokines, TNFα, IL-1 and IL-6	MPAK signalling	5 µM	[95]
	Hippocampal neuronal cells HT-24	↑ Antiapoptotic factors ↓ Proapoptotic factors ↓ Phosphorylation of dynamin-related protein 1 (Drp1)	Apoptosis	10 µM/L	[96]
	Lead poisoned Kunming mice	↑ GPX, SOD, CAT and GSH	antioxidant defence system	0.1, 1.0, 10.0 mg/kg	[97]
	Male C57BL mice	↓ TNF- α, IL-1	NF κB and P50/P65 pathway	0.1 mg/kg, 1 mg/kg	[98]
	Male CD1 mice	↓ NALP3, ASC, caspase-1, and IL-1β	NALP3 inflammasome	0.1 mg/kg, 1 mg/kg	[99]

**Abbreviations***:***AIF**, apoptosis-inducing factor; **Apaf-1**, apoptotic peptidase activating factor 1; **ASC**, alanine-serine-cysteine; **CAT**, catalase; **COX-2**, cyclooxygenase-2; **Endo G**, endonuclease G; **ERK**, extracellular signal-regulated kinase; **GLUT 4**, glucose transporter 4; **GPX**, glutathione peroxidase; **GSH**, glutathione; **GST**, glutathione-S-transferase; **IL-6**, interleukin-6; **iNOS**, inducible nitric oxide synthase; **JNK**, Jun N-terminal Kinase; **LDH**, lactate dehydrogenase; **LPS**, lipopolysaccharide; **MLP-1**, muscle LIM-domain protein; **MPAK**, mitogen-activated protein kinase; **NALP3**, NACHT, LRR and PYD domains-containing protein 3; **NF-κB**, nuclear factor kappa-light-chain-enhancer of activated B cells; **P-38**, mitogen-activated protein kinases; **P-AKT**, phospho- phosphatidylinositol-3-kinase; **P-EGFR**, phospho epidermal growth factor receptor; **PGE2**, prostaglandin E2; **P-MTOR**, phospho-mammalian target of rapamycin; **PPAR-γ**, peroxisome proliferator-activated receptor gamma; **PTP1B**, epithelial protein-tyrosine phosphatase 1B; **RIP1**, receptor interacting protein; **SOD**, superoxide dismutase; **STAT3-P**, signal transducer and activator of transcription 3 phosphorylated; **TNF-α**, tumor necrosis factor-alpha.

**Table 3:** Minimum inhibitory concentrations (MIC) of chrysophanol against microorganism.

Microbes	MIC (μg/mL)	References
*Bacillus cereus*	>250	[76]
*B. subtilis*	250	[76]
*Staphylococcus aureus*	>250	[76]
*S. epidermidis*	31.25	[76]
*Staphylococcus warneri*	>128	[76]
*Escherichia coli*	125	[76]
*Shigella sonnei*	>250	
*Aeromonas* *hydrophila IB101*	200	[120]
*A. hydrophila JG101*	200	[120]
*A. hydrophila TPS-30*	200	[120]
*A. hydrophila BSK-10*	200	[120]
*A. hydrophila 4LNS301*	200	[120]
*A. hydrophila CCH201*	200	[120]
*A. hydrophila LNB101*	200	[120]
*A. hydrophila CG101*	200	[120]
*Micrococcus kristinae*	>250	[43]
*Proteus vulgaris*	125, 128	[43]
*Enterobacter aerogenes*	>250	[43]
*Pseudomonas aeruginosa*	128	[72]
*Vibrio harveyi*	1000	[121]
*Candida albicans*	50, 128	[122]
*Cryptococcus neoformans*	50	[122]
*Aspergillus fumigatus*	50	[122]
*Trichophyton mentagrophytes*	25, 1250	[76,122]
*T. rubrum*	156	[76]
*Epidermophyton floccosum*	625	[76]
